# Supplementary material for: Lexical markers of disordered speech in primary progressive aphasia and ‘Parkinson-plus’ disorders
Source: Brain Commun. 2024 Nov 29;6(6):fcae433. doi: 10.1093/braincomms/fcae433 (PMC11630745; doi:10.1093/braincomms/fcae433)
Supplement: fcae433_Supplementary_Data [file fcae433_supplementary_data.pdf]

Appendix: Cambridge Language List (CALL)

The scoresheets using the 15 words are shown below in Appendices 1 and 2. The first column shows the 15 target words. In the second column, the examiner marks if the participant produced each specific word or not. While variations in morpho-syntactic word forms (e.g., wearing instead of wear/worn) should be marked as correct, synonyms and related words (e.g., "woman" for "lady") should not. The third column shows the specific "points" associated with each of the words that help to differentiate between healthy controls and patients. The "points" per word were derived directly from the LASSO coefficients. To make CALL easy to use, we rounded up the coefficient and cut-off values to 1 decimal point and then multiplied all values by ten.

As an example, for the BDAE 'cookie theft' picture, production of the word "doing" is credited with 8 "points", whilst the production of "something" is debited with 6 "penalty points". The total points for the words that each participant produced are calculated. If the summed value exceeds 60 then a "diagnosis" of control is more likely; a score below 60 denotes that the participant is more likely to be a patient. If an indicative diagnosis of patient results (from the scoring of column 3), then a similar secondary scoring process is undertaken. This time, the fourth column provides the positive (blue) and negative (red) "points" associated with the prediction of "lexico-semantic" vs. "motor" patient group membership. Again, the total positive and negative points for the words that each participant produced are calculated. If the summed value exceeds 21 then a "diagnosis" of "lexico-semantic" patient is more likely; a score below 21 denotes that the participant is more likely to be a "motor" patient. For a worked example of a representative svPPA patient, see Supplementary Table 9.

Appendix 1

Please see below the checklist scoresheets for the (A) BDAE 'cookie theft' picture narrative and (B) the MLSE 'beach scene' picture narrative differentiating healthy controls versus patients, and between "lexico-semantic" and "motor" groups.

**(A) Cookie Theft Picture Narrative: Scoresheet for controls versus patients and "lexico-semantic" versus "motor" groups**			
**Step 1**: Listen for these words in the participant's narrative	**Step 2**: Score - was each word produced by the participant at least once?<br>✓ - yes ✗ - no	**Step 3**: Cross-out the points if the word was not produced	**Step 4**: Cross-out the points if the word was not produced
Doing		8	n/a
Drying		19	
Over		3	
Garden		5	
Overflow		29	-15
Stool		15	-11
Open		19	-5
Sink		4	-4
Not		3	19
Water		1	7
Something		-6	2
Has/have		n/a	16
Little			11
Lady			14
Looking			n/a
**Step 5**: Sum the positive and negative "points" *only* for the words produced:		**Total score =** ________	**Total score =** ________
**Interpretation**		Control vs Patient	Motor vs Lexico-Semantic Patient
		"Control" if the total score is greater than 60 and "patient" if it is less than 60	"Lexico-semantic" if the total score is greater than 21 and "motor" if it is less than 21

(B) Beach Scene Picture Narrative: Scoresheet for controls versus patients and "lexico-semantic" versus "motor" groups			
**Step 1**: Listen for these words in the participant's narrative	**Step 2**: Score - was each word produced by the participant at least once?<br>✓ - yes ✗ - no	**Step 3**: Cross-out the points if the word was not produced	**Step 4**: Cross-out the points if the word was not produced
Seagull		n/a	-10
Sandcastle		4	-2
Book		4	-4
Sun		19	n/a
Dig		11	
Bone		13	
Wearing		23	
Sand		2	
Sea		6	
Towel		3	
Reading		n/a	
Beach			4
Know			13
Got			16
Do			12
**Step 5**: Sum the positive and negative "points" *only* for the words produced:		**Total score =** ________	**Total score =** ________
**Interpretation**		Control vs Patient	Motor vs Lexico-Semantic Patient
		"Control" if the total score is greater than 53 and "patient" if it is less than 53	"Lexico-semantic" if the total score is greater than 13 and "motor" if it is less than 13

Appendix 2

The checklist scoresheets for additional diagnostic differentiations. As in the main manuscript, we employed a hierarchical classification (i.e., controls versus patients; “lexico-semantic” versus “motor” groups) to the checklists as shown in Appendix 1 as the LASSO regressions for svPPA versus lvPPA, and nfvPPA versus PSP resulted in zero words for both pictures. Under each checklist below, we provide the within-group and out-of-sample validation accuracies to use for reference.

**Cookie Theft Picture Narrative: Scoresheet for svPPA versus nfvPPA, PSP, and CBS**				
**Step 1**: Listen for these words in the participant’s narrative	**Step 2**: Score - was each word produced by the participant at least once?<br>✓ - yes ✗ - no	**Step 3**: Cross-out the points if the word was not produced		
Something		n/a	n/a	n/a
Drying				
Overflow				-50
Stool				-24
Little				-3
Water			4	24
Not		11	18	20
Lady		57	43	27
Garden		26	17	n/a
Looking		31	16	
Doing		28	15	
Sink		-4	-8	5
Open		-1	n/a	n/a
Over		n/a		5
Has/have				13
**Step 4**: Sum the positive and negative “points” *only* for the words produced:		**Total score =** ______	**Total score =** ______	**Total score =** ______
**Interpretation**		svPPA vs nfvPPA	svPPA vs PSP	svPPA vs CBS
		“svPPA” if the total score is greater than 33 and “nfvPPA” if it is less than 33	“svPPA” if the total score is greater than 30 and “PSP” if it is less than 30	“svPPA” if the total score is greater than 21 and “CBS” if it is less than 21

Note: For the BDAE ‘cookie theft’ picture, the within-sample four-fold classification and out-of-sample validation accuracies were: (i) 100% and 67% between svPPA versus nfvPPA patients; (ii) 100% and 78% between svPPA versus PSP patients; and (iii) 100% and 92% between svPPA versus CBS patients, respectively.

<table>
<tr><th colspan="5">Beach Scene Picture Narrative: Scoresheet for svPPA versus nfvPPA, PSP, and CBS</th></tr>
<tr><td>Step 1: Listen for these words in the participant's narrative</td><td>Step 2: Score - was each word produced by the participant at least once?
✓ - yes × - no</td><td colspan="3">Step 3: Cross-out the points if the word was not produced</td></tr>
<tr><td>Know</td><td></td><td>5</td><td>n/a</td><td>8</td></tr>
<tr><td>Got</td><td></td><td>11</td><td>7</td><td>6</td></tr>
<tr><td>Do</td><td></td><td>18</td><td>27</td><td>23</td></tr>
<tr><td>Seagull</td><td></td><td>-1</td><td>-15</td><td>-9</td></tr>
<tr><td>Sandcastle</td><td></td><td>-11</td><td>-22</td><td rowspan="11">n/a</td></tr>
<tr><td>Towel</td><td></td><td rowspan="10">n/a</td><td>-8</td></tr>
<tr><td>Beach</td><td></td><td>17</td></tr>
<tr><td>Book</td><td></td><td rowspan="8">n/a</td></tr>
<tr><td>Sun</td><td></td></tr>
<tr><td>Dig</td><td></td></tr>
<tr><td>Bone</td><td></td></tr>
<tr><td>Wearing</td><td></td></tr>
<tr><td>Sand</td><td></td></tr>
<tr><td>Sea</td><td></td></tr>
<tr><td>Reading</td><td></td></tr>
<tr><td colspan="2">Step 4: Sum the positive and negative "points" <u>only</u> for the words produced:</td><td>Total score = ________</td><td>Total score = ________</td><td>Total score = ________</td></tr>
<tr><td colspan="2" rowspan="2">Interpretation</td><td>svPPA vs nfvPPA</td><td>svPPA vs PSP</td><td>svPPA vs CBS</td></tr>
<tr><td>"svPPA" if the total score is greater than 7 and "nfvPPA" if it is less than 7</td><td>"svPPA" if the total score is greater than 13 and "PSP" if it is less than 13</td><td>"svPPA" if the total score is greater than 12 and "CBS" if it is less than 12</td></tr>
</table>

Note: For the MLSE 'beach scene' picture, the within-sample four-fold classification and out-of-sample validation accuracies were: (i) 94% and 50% between svPPA versus nfvPPA patients; (ii) 100% and 56% between svPPA versus PSP patients; and (iii) 95% and 69% between svPPA versus CBS patients, respectively.

<table>
<tr><th colspan="5">Cookie Theft Picture Narrative: Scoresheet for lvPPA versus nfvPPA, PSP, and CBS</th></tr>
<tr><td>Step 1: Listen for these words in the participant's narrative</td><td>Step 2: Score - was each word produced by the participant at least once?
✓ - yes ✗ - no</td><td colspan="3">Step 3: Cross-out the points if the word was not produced</td></tr>
<tr><td>Drying</td><td></td><td rowspan="2">n/a</td><td>-9</td><td rowspan="2">n/a</td></tr>
<tr><td>Lady</td><td></td><td>5</td></tr>
<tr><td>Has/have</td><td></td><td>1</td><td>19</td><td>21</td></tr>
<tr><td>Little</td><td></td><td>13</td><td>6</td><td>16</td></tr>
<tr><td>Not</td><td></td><td>13</td><td rowspan="11">n/a</td><td>11</td></tr>
<tr><td>Open</td><td></td><td>-1</td><td rowspan="3">n/a</td></tr>
<tr><td>Sink</td><td></td><td>-11</td></tr>
<tr><td>Water</td><td></td><td rowspan="8">n/a</td></tr>
<tr><td>Something</td><td></td><td>3</td></tr>
<tr><td>Looking</td><td></td><td>-8</td></tr>
<tr><td>Overflow</td><td></td><td>-7</td></tr>
<tr><td>Stool</td><td></td><td>-27</td></tr>
<tr><td>Doing</td><td></td><td rowspan="3">n/a</td></tr>
<tr><td>Over</td><td></td></tr>
<tr><td>Garden</td><td></td></tr>
<tr><td colspan="2">Step 4: Sum the positive and negative "points" only for the words produced:</td><td>Total score = ________</td><td>Total score = ________</td><td>Total score = ________</td></tr>
<tr><td colspan="2" rowspan="2">Interpretation</td><td>lvPPA vs nfvPPA</td><td>lvPPA vs PSP</td><td>lvPPA vs CBS</td></tr>
<tr><td>"lvPPA" if the total score is greater than 6 and "nfvPPA" if it is less than 6</td><td>"lvPPA" if the total score is greater than 11 and "PSP" if it is less than 11</td><td>"lvPPA" if the total score is greater than 8 and "CBS" if it is less than 8</td></tr>
</table>

Note: For the BDAE 'cookie theft' picture, the within-sample four-fold classification and out-of-sample validation accuracies were: (i) 94% and 63% between lvPPA versus nfvPPA patients; (ii) 89% and 63% between lvPPA versus PSP patients; and (iii) 100% and 55% between lvPPA versus CBS patients, respectively.

<table>
<tr><th colspan="5">Beach Scene Picture Narrative: Scoresheet for lvPPA versus nfvPPA, PSP, and CBS</th></tr>
<tr><td>Step 1: Listen for these words in the participant's narrative</td><td>Step 2: Score - was each word produced by the participant at least once?
✓ - yes ✗ - no</td><td colspan="3">Step 3: Cross-out the points if the word was not produced</td></tr>
<tr><td>Bone</td><td></td><td>-3</td><td colspan="2" rowspan="2">n/a</td></tr>
<tr><td>Reading</td><td></td><td>-1</td></tr>
<tr><td>Seagull</td><td></td><td>-7</td><td>-5</td><td>-11</td></tr>
<tr><td>Know</td><td></td><td>9</td><td>4</td><td>6</td></tr>
<tr><td>Sand</td><td></td><td>7</td><td>1</td><td rowspan="3">n/a</td></tr>
<tr><td>Towel</td><td></td><td>10</td><td>n/a</td></tr>
<tr><td>Beach</td><td></td><td rowspan="9">n/a</td><td>14</td></tr>
<tr><td>Book</td><td></td><td rowspan="8">n/a</td><td>-2</td></tr>
<tr><td>Got</td><td></td><td>2</td></tr>
<tr><td>Do</td><td></td><td>2</td></tr>
<tr><td>Sandcastle</td><td></td><td rowspan="5">n/a</td></tr>
<tr><td>Sun</td><td></td></tr>
<tr><td>Dig</td><td></td></tr>
<tr><td>Wearing</td><td></td></tr>
<tr><td>Sea</td><td></td></tr>
<tr><td colspan="2">Step 4: Sum the positive and negative "points" <u>only</u> for the words produced:</td><td>Total score = ________</td><td>Total score = ________</td><td>Total score = ________</td></tr>
<tr><td colspan="2" rowspan="2">Interpretation</td><td>lvPPA vs nfvPPA</td><td>lvPPA vs PSP</td><td>lvPPA vs CBS</td></tr>
<tr><td>"lvPPA" if the total score is greater than 3 and "nfvPPA" if it is less than 3</td><td>"lvPPA" if the total score is greater than 8 and "PSP" if it is less than 8</td><td>"lvPPA" if the total score is greater than 2 and "CBS" if it is less than 2</td></tr>
</table>

Note: For the MLSE 'beach scene' picture, the within-sample four-fold classification and out-of-sample validation accuracies were: (i) 88% and 63% between lvPPA versus nfvPPA patients; (ii) 78% and 31% between lvPPA versus PSP patients; and (iii) 81% and 55% between lvPPA versus CBS patients, respectively.

**Cookie Theft Picture Narrative: Scoresheet for CBS versus nfvPPA and PSP**			
**Step 1**: Listen for these words in the participant's narrative	**Step 2**: Score - was each word produced by the participant at least once?<br>✓ - yes ✗ - no	**Step 3**: Cross-out the points if the word was not produced	
Open		-1	n/a
Sink		-11	
Not		13	
Has/have		1	
Little		13	
Garden		n/a	11
Stool			2
Lady			5
Looking			4
Doing			n/a
Drying			
Over			
Overflow			
Water			
Something			
**Step 4**: Sum the positive and negative "points" *only* for the words produced:		**Total score =** ________	**Total score =** ________
**Interpretation**		CBS vs nfvPPA	CBS vs PSP
		"CBS" if the total score is greater than 6 and "nfvPPA" if it is less than 6	"CBS" if the total score is greater than 3 and "PSP" if it is less than 3

Note: For the BDAE 'cookie theft' picture, the within-sample four-fold classification and out-of-sample validation accuracies were: (i) 95% and 45% between CBS versus nfvPPA patients; and (iii) 78% and 63% between CBS versus PSP patients, respectively.

Beach Scene Picture Narrative: Scoresheet for CBS versus nfvPPA and PSP			
**Step 1**: Listen for these words in the participant's narrative	**Step 2**: Score - was each word produced by the participant at least once?<br>✓ - yes ✗ - no	**Step 3**: Cross-out the points if the word was not produced	
Sand		22	n/a
Towel		18	
Beach		5	5
Book		4	2
Sandcastle		-5	n/a
Sun		-1	
Reading		n/a	2
Seagull			4
Bone			n/a
Know			
Got			
Do			
Dig			
Wearing			
Sea			
**Step 4**: Sum the positive and negative "points" *only* for the words produced:		**Total score =** ________	**Total score =** ________
**Interpretation**		CBS vs nfvPPA	CBS vs PSP
		"CBS" if the total score is greater than 3 and "nfvPPA" if it is less than 3	"CBS" if the total score is greater than 3 and "PSP" if it is less than 3

Note: For the MLSE 'beach scene' picture, the within-sample four-fold classification and out-of-sample validation accuracies were: (i) 86% and 73% between CBS versus nfvPPA patients; and (iii) 78% and 73% between CBS versus PSP patients, respectively.

Supplementary Material

Supplementary Table 1 Loadings for principal component analysis of quantitative measures of speech fluency

Measure	PC 1 (“Speech Quanta”)	PC 2 (“Lexical Richness”)	PC 3 (“Speech Complexity”)
Number of Words	**0.97**	-0.21	0.00
Number of Word Bigrams	**0.97**	-0.21	0.00
Number of Word Trigrams	**0.97**	-0.20	0.00
Type of Words	**0.97**	0.00	0.00
Type of Word Bigrams	**0.98**	-0.14	0.00
Type of Word Trigrams	**0.98**	-0.16	0.00
Combination Ratio	**0.66**	0.00	**0.52**
Word Per Minute	**0.60**	0.00	**0.72**
Total Time	0.48	-0.11	**-0.81**
TTR of Words	**-0.67**	**0.59**	0.00
TTR of Word Bigrams	-0.32	**0.91**	0.00
TTR of Word Trigrams	0.00	**0.93**	0.00
Proportion of Function Words	0.40	-0.19	0.29

Rotation: Orthogonal varimax. Loadings above a threshold of 0.5 are bolded. PC, principal component; TTR, type-to-token ratio.

Supplementary Table 2 Correlations between Mini Linguistic State Examination (MLSE) and principal component (PC) scores

	MLSE Motor speech	MLSE Syntax	MLSE Semantics	MLSE Phonology	MLSE Working Memory	MLSE Total
**Speech fluency PCA**						
PC 1 'speech quanta'						
All groups	**R = 0.54, p < 0.001**	**R = 0.42, p < 0.001**	R = 0.11, p = 0.38	**R = 0.5, p < 0.001**	R = 0.08, p = 0.51	**R = 0.45, p < 0.001**
Controls	R = -0.08, p = 0.71	R = 0.29, p = 0.16	R = -0.15, p = 0.47	R = 0.23, p = 0.28	R = -0.16, p = 0.45	R = 0.09, p = 0.69
svPPA	R = 0.23, p = 0.59	R = -0.21, p = 0.61	R = 0.23, p = 0.59	R = 0.38, p = 0.36	R = -0.65, p = 0.08	R = -0.03, p = 0.94
lvPPA	**R = 0.83, p = 0.02**	R = 0.44, p = 0.33	R = 0.62, p = 0.14	**R = 0.94, p = 0.002**	R = -0.23, p = 0.62	**R = 0.88, p = 0.009**
nfvPPA	R = -0.27, p = 0.52	R = -0.01, p = 0.99	R = 0.41, p = 0.32	R = -0.3, p = 0.48	R = -0.16, p = 0.71	R = -0.19, p = 0.65
PSP	R = 0.12, p = 0.75	R = -0.17, p = 0.65	R = 0.44, p = 0.24	R = 0.3, p = 0.44	R = -0.02, p = 0.96	R = 0.09, p = 0.81
CBS	R = 0.49, p = 0.15	R = 0.31, p = 0.38	R = -0.35, p = 0.32	R = 0.16, p = 0.65	R = -0.51, p = 0.13	R = 0.13, p = 0.71
PC 2 'lexical richness'						
All groups	R = -0.16, p = 0.21	R = 0.05, p = 0.68	**R = 0.44, p < 0.001**	R = 0.03, p = 0.8	R = -0.01, p = 0.92	R = 0.14, p = 0.27
Controls	R = 0.13, p = 0.53	R = -0.11, p =0.6	R = -0.07, p = 0.73	R = -0.35, p = 0.10	R = -0.37, p = 0.07	R = -0.36, p = 0.09
svPPA	R = 0.02, p = 0.97	R = 0.15, p = 0.73	R = -0.31, p = 0.46	R = 0.06, p = 0.89	R = -0.17, p = 0.69	R = -0.19, p = 0.66
lvPPA	R = 0.66, p = 0.1	R = 0.45, p = 0.31	R = 0.73, p = 0.06	**R = 0.97, p < 0.001**	R = -0.3, p = 0.52	**R = 0.96, p < 0.001**
nfvPPA	R = -0.58, p = 0.13	R = -0.28, p = 0.5	R = 0.38, p = 0.34	R = -0.37, p = 0.36	R = -0.5, p = 0.21	R = -0.44, p = 0.27
PSP	R = 0.34, p = 0.37	R = 0.22, p = 0.56	R = 0.12, p = 0.75	R = 0.47, p = 0.21	R = 0.11, p = 0.77	R = 0.4, p = 0.29
CBS	R = -0.29, p = 0.42	R = 0.16, p = 0.67	R = 0.25, p = 0.48	R = 0.12, p = 0.74	R = 0.52, p = 0.12	R = 0.09, p = 0.81
PC 3 'speech complexity'						
All groups	**R = 0.49, p < 0.001**	**R = 0.52, p < 0.001**	**R = 0.29, p = 0.02**	**R = 0.53, p < 0.001**	**R = 0.5, p < 0.001**	**R = 0.62, p < 0.001**
Controls	R = 0.34, p = 0.1	R = -0.03, p = 0.89	R = -0.09, p = 0.67	R = 0.1, p = 0.63	**R = 0.49, p = 0.01**	R = 0.28, p = 0.18
svPPA	R = -0.35, p = 0.4	R = -0.18, p = 0.66	R = -0.17, p = 0.7	R = 0.14, p = 0.74	R = 0.04, p = 0.93	R = -0.12, p = 0.79
lvPPA	R = -0.02, p = 0.97	R = -0.33, p = 0.47	R = -0.25, p = 0.59	R = -0.53, p = 0.22	R = -0.02, p = 0.97	R = -0.41, p = 0.36
nfvPPA	R = 0.36, p = 0.38	R = 0.69, p = 0.06	R = 0.62, p = 0.10	**R = 0.9, p = 0.003**	R = 0.63, p = 0.09	**R = 0.77, p = 0.03**
PSP	**R = 0.88, p = 0.002**	R = 0.34, p = 0.37	R = 0.37, p = 0.33	R = 0.34, p = 0.36	R = 0.3, p = 0.43	**R = 0.75, p = 0.02**
CBS	**R = 0.66, p = 0.04**	R = 0.44, p = 0.21	**R = 0.83, p = 0.003**	**R = 0.88, p < 0.001**	R = 0.51, p = 0.13	**R = 0.91, p < 0.001**
**Word Properties PCA**						
PC 1 'length'						
All groups	R = 0.08, p = 0.53	R = 0.14, p = 0.26	**R = 0.56, p < 0.001**	**R = 0.24, p = 0.05**	**R = 0.26, p = 0.03**	**R = 0.38, p = 0.001**
Controls	R = -0.07, p = 0.74	R = -0.26, p = 0.21	R = -0.16, p = 0.46	R = 0.02, p = 0.94	R = -0.21, p = 0.32	R = -0.17, p = 0.42
svPPA	R = -0.29, p = 0.49	R = -0.38, p = 0.35	R = -0.04, p = 0.92	R = -0.64, p = 0.09	R = 0.18, p = 0.68	R = -0.33, p = 0.43
lvPPA	R = 0.38, p = 0.36	R = -0.34, p = 0.41	R = 0.62, p = 0.10	R = 0.6, p = 0.12	R = -0.06, p = 0.89	R = 0.59, p = 0.13
nfvPPA	R = -0.05, p = 0.9	R = 0.11, p = 0.79	R = -0.32, p = 0.41	R = 0.07, p = 0.85	R = 0.13, p = 0.74	R = 0.04, p = 0.91
PSP	R = -0.4, p = 0.29	R = -0.24, p = 0.54	R = -0.26, p = 0.49	R = -0.09, p = 0.81	R = -0.36, p = 0.34	R = -0.34, p = 0.38
CBS	R = 0.43, p = 0.21	R = -0.04, p = 0.91	**R = 0.63, p = 0.05**	R = 0.58, p = 0.08	R = 0.3, p = 0.4	R = 0.57, p = 0.09
PC 2 'semantic richness'						
All groups	**R = 0.26, p = 0.03**	R = -0.08, p = 0.52	**R = -0.54, p < 0.001**	R = 0.17, p = 0.16	R = -0.21, p = 0.09	R = -0.15, p = 0.23

Controls	R = -0.06, *p* = 0.78	R = 0.16, *p* = 0.45	R = 0.26, *p* = 0.21	R = 0.1, *p* = 0.63	R = -0.31, *p* = 0.15	R = -0.002, *p* = 0.99
svPPA	R = -0.10, *p* = 0.82	R = -0.64, *p* = 0.09	R = -0.33, *p* = 0.42	R = 0.33, *p* = 0.43	**R = -0.76, *p* = 0.03**	R = -0.58, *p* = 0.13
lvPPA	R = 0.65, *p* = 0.08	R = -0.10, *p* = 0.82	R = -0.03, *p* = 0.94	R = 0.62, *p* = 0.1	R = 0.11, *p* = 0.79	R = 0.28, *p* = 0.5
nfvPPA	R = -0.09, *p* = 0.82	R = 0.07, *p* = 0.86	R = 0.03, *p* = 0.93	R = 0.3, *p* = 0.43	R = -0.007, *p* = 0.98	R = 0.09, *p* = 0.81
PSP	R = 0.03, *p* = 0.94	R = -0.63, *p* = 0.07	R = 0.07, *p* = 0.86	R = -0.07, *p* = 0.85	R = -0.23, *p* = 0.55	R = -0.16, *p* = 0.68
CBS	R = 0.06, *p* = 0.88	R = 0.59, *p* = 0.08	R = -0.13, *p* = 0.73	R = -0.004, *p* = 0.99	R = 0.02, *p* = 0.96	R = 0.08, *p* = 0.83
PC 3 'acquisition age'						
All groups	**R = 0.55, *p* < 0.001**	R = 0.23, *p* = 0.06	R = -0.09, *p* = 0.46	**R = 0.53, *p* < 0.001**	R = 0.09, *p* = 0.46	**R = 0.33, *p* = 0.007**
Controls	R = 0.07, *p* = 0.74	R = 0.34, *p* = 0.1	R = 0.02, *p* = 0.94	R = -0.11, *p* = 0.59	R = -0.34, *p* = 0.1	R = -0.12, *p* = 0.59
svPPA	R = 0.08, *p* = 0.85	R = -0.2, *p* = 0.64	R = -0.22, *p* = 0.61	R = 0.42, *p* = 0.3	R = -0.14, *p* = 0.74	R = -0.09, *p* = 0.84
lvPPA	**R = 0.84, *p* = 0.009**	R = 0.11, *p* = 0.8	R = 0.4, *p* = 0.32	**R = 0.86, *p* = 0.006**	R = -0.16, *p* = 0.7	R = 0.6, *p* = 0.12
nfvPPA	R = 0.37, *p* = 0.32	R = -0.29, *p* = 0.44	R = -0.12, *p* = 0.75	R = 0.28, *p* = 0.47	R = 0.3, *p* = 0.44	R = 0.23, *p* = 0.55
PSP	R = -0.22, *p* = 0.57	R = -0.31, *p* = 0.41	R = -0.02, *p* = 0.97	R = 0.45, *p* = 0.23	R = 0.27, *p* = 0.48	R = -0.008, *p* = 0.98
CBS	R = 0.57, *p* = 0.08	**R = 0.71, *p* = 0.02**	R = 0.41, *p* = 0.24	R = 0.55, *p* = 0.1	R = 0.16, *p* = 0.66	**R = 0.65, *p* = 0.04**

Note: Significant correlations are indicated in bold font. CBS, corticobasal syndrome; lvPPA, logopenic variant primary progressive aphasia; nfvPPA, non-fluent variant primary progressive aphasia; PCA, principal component analysis; PSP, progressive supranuclear palsy; svPPA, semantic variant primary progressive aphasia.

Supplementary Table 3 Loadings for principal component analysis of quantitative measures of word properties

Measure	PC 1 (“Length”)	PC 2 (“Semantic richness”)	PC 3 (“Acquisition age”)
Length	**0.89**	-0.14	0.22
OLD	**0.95**	0.00	0.19
PLD	**0.94**	0.00	0.16
Log Frequency	-0.28	**0.88**	-0.22
Semantic Diversity	0.00	**0.86**	0.23
SND	-0.19	**0.84**	-0.30
Concreteness	-0.18	**-0.65**	**-0.59**
Age of Acquisition	0.35	-0.17	**0.81**

Rotation: Orthogonal varimax. Loadings above a threshold of 0.5 are bolded. OLD, orthographic Levenshtein distance; PC, principal component; PLD, phonological Levenshtein distance; SND, semantic neighbourhood density.

Supplementary Table 4 Distribution analysis. ANOVA findings on the effects of group, quartile and group-by-quartile interaction from the distribution analysis of word properties principal component analysis

**Principal Component (PC)**	**Task**	**ANOVA**	**Tukey's HSD Test for multiple comparison**
**PC 1 ('Length')**	BDAE 'cookie theft'	Effect of group only: ($F(5,283) = 37.16$, $p < 0.001$)	Controls > all patients ($p < 0.001$), svPPA > nfvPPA, PSP and CBS ($p < 0.01$), lvPPA > nfvPPA ($p = 0.005$)
	MLSE 'beach scene'	Effect of group only: ($F(5,272) = 39.18$, $p < 0.001$)	Controls > all patients ($p < 0.001$), svPPA > nfvPPA, PSP and CBS ($p \leq 0.001$), lvPPA and CBS > nfvPPA ($p < 0.05$)
**PC 2 ('Semantic richness')**	BDAE 'cookie theft'	Effects of group ($F(5,280) = 33.68$, $p < 0.001$), quartile ($F(1,280) = 4.67$, $p = 0.03$), and group-by-quartile interaction ($F(5,280) = 4.36$, $p < 0.001$)	For group: Controls > all patients ($p < 0.001$), svPPA > nfvPPA, PSP and CBS ($p < 0.005$), lvPPA > nfvPPA ($p < 0.02$)<br><br>For quartile: first > second ($p = 0.05$), third > second ($p = 0.02$), fourth > second ($p < 0.001$)
	MLSE 'beach scene'	Effects of group ($F(5,270) = 28.94$, $p < 0.001$), quartile ($F(1,270) = 5.53$, $p = 0.02$), and group-by-quartile interaction ($F(5,270) = 8.29$, $p < 0.001$).	For group: Controls > all patients ($p \leq 0.005$), svPPA > nfvPPA, PSP and CBS ($p < 0.01$), lvPPA > nfvPPA ($p < 0.001$)<br><br>For quartile: second > first ($p = 0.007$), second > third ($p = 0.007$), second > fourth ($p < 0.001$)
**PC 3 ('Acquisition Age)**	BDAE 'cookie theft'	Effects of group ($F(5,283) = 36.15$, $p < 0.001$), quartile ($F(1,283) = 17.17$, $p < 0.001$), and group-by-quartile interaction ($F(5,283) = 2.47$, $p = 0.03$)	For group: Controls > all patients ($p < 0.001$), svPPA > nfvPPA, PSP and CBS ($p < 0.01$), lvPPA > nfvPPA ($p < 0.005$)<br><br>For quartile: third > first ($p = 0.01$), fourth > first ($p = 0.01$), third > second ($p = 0.002$), fourth > second ($p = 0.002$)
	MLSE 'beach scene'	Effects of group ($F(5,265) = 31.04$, $p < 0.001$), quartile ($F(1,265) = 21.67$, $p < 0.001$), and group-by-quartile interaction ($F(5,265) = 2.47$, $p = 0.03$)	For group: Controls > all patients ($p \leq 0.007$), svPPA > nfvPPA, PSP and CBS ($p < 0.01$), lvPPA > nfvPPA ($p < 0.001$)<br><br>For quartile: first > third ($p = 0.01$), first > fourth ($p < 0.001$), second > third ($p < 0.001$), second > fourth ($p < 0.001$)

CBS, corticobasal syndrome; lvPPA, logopenic variant primary progressive aphasia; nfvPPA, non-fluent variant primary progressive aphasia; PSP, progressive supranuclear palsy; svPPA, semantic variant primary progressive aphasia.

Supplementary Table 5 VBM results in the whole group. Voxel based morphometry results showing regions of grey matter intensity that correlate with PCA-generated principal component in the whole group

Principal Component	Regions	Hemisphere	Number of Voxels	Peak MNI coordinates			t-value
**Speech quanta (Supplementary Table 1 PC 1)**	Middle and superior frontal gyri	Left	407	-22	22	48	5.56
	Middle and superior frontal gyri and supplementary motor area	Right	287	18	26	58	4.25
	Inferior frontal gyrus and insula	Right	235	36	24	6	4.95
	Putamen and caudate	Right	229	20	14	0	5.54
**Speech complexity (Supplementary Table 1 PC 3)**	Insula, inferior frontal gyrus, extending into the superior temporal gyrus	Left	1405	-44	6	4	5.65
	Medial frontal gyrus, superior frontal gyrus, and anterior cingulate	Left	245	-4	54	-2	4.97
	Middle and superior frontal gyri	Left	115	-24	34	44	4.21
	Parahippocampal gyrus, amygdala and hippocampus	Left	109	-26	-10	-12	4.24
**Length (Supplementary Table 3 PC 1)**	Insula, middle and superior temporal gyri	Left	828	-44	-6	-8	5.32
	Parahippocampal and fusiform gyri	Left	356	-24	-34	-20	4.99
	Limbic lobe, including the anterior cingulate and caudate	Right	236	4	12	-10	4.45
	Inferior and middle temporal gyri and fusiform gyri	Right	200	46	-10	-38	4.52
	Parahippocampal gyrus, hippocampus, fusiform and amygdala	Right	139	30	-12	-32	4.05
**Acquisition age (Supplementary Table 3 PC 3)**	Cingulate gyrus	Bilateral	196	2	-8	44	4.35
	Caudate and putamen	Right	102	16	14	6	4.12

PCA, principal component analysis.

Supplementary Table 6 VBM results in patients. Voxel based morphometry results showing regions of grey matter intensity that correlate with PCA-generated factors in patients only

Principal Component	Regions	Hemisphere	Number of Voxels	Peak MNI coordinates			t-value
**Length (Supplementary Table 3 PC 1)**	Insula, middle and superior temporal gyri	Left	184	-46	-8	-6	4.69

PCA, principal component analysis.

Supplementary Table 7 VBM results with cluster-forming height threshold. Voxel based morphometry results showing regions of grey matter intensity that correlate with PCA-generated factors with a cluster-forming height threshold of $p < 0.005$ paired with a cluster extent threshold of $p < 0.05$ FWE-corrected

**Principal Component**	**Regions**	**Hemisphere**	**Number of Voxels**	**Peak MNI coordinates**			**t-value**
**Speech complexity (Supplementary Table 1 PC 3)**	Insula, inferior frontal gyrus, extending into the superior temporal gyrus	Left	1405	-44	6	4	5.65
**Length (Supplementary Table 3 PC 1)**	Insula, middle and superior temporal gyri	Left	828	-44	-6	-8	5.32

PCA, principal component analysis.

Supplementary Table 8 LASSO results comparing all patients versus controls, "lexico-semantic" (svPPA and lvPPA) versus "motor" (nfvPPA, PSP, and CBS) groups, svPPA versus lvPPA patients, and nfvPPA and PSP versus CBS patients

	**Word checklist: LASSO value**	**Word checklist with cognitive scores: LASSO value**
	**1. BDAE 'cookie theft'**	
**Controls versus patients**		
Model intercept	-5.99	-7.76
Overflow	2.94	0.26
Stool	1.53	
Open	1.92	0.44
Not	0.25	
Water	0.14	
Sink	0.39	0.27
Doing	0.81	0.31
Something	-0.60	
Drying	1.91	1.92
Over	0.33	0.02
Garden	0.45	0.37
MLSE: Syntax		0.01
MLSE: Working memory		0.07
ACE-R: Fluency		0.55
**"Motor" (nfvPPA, PSP, CBS) versus "Lexico-semantic" (svPPA, lvPPA)**		
Model intercept	-2.07	1.91
Overflow	-1.47	
Stool	-1.11	-0.23
Open	-0.53	
Not	1.92	1.09
Water	0.72	
Sink	-0.40	
Something	0.17	
Has/have	1.64	0.69
Little	1.07	0.91
Lady	1.40	
MLSE: Motor speech		0.02
MLSE: Semantics		-0.27
**svPPA versus lvPPA**		
Model intercept	NA	-1.56
Drying		1.12
MLSE: Semantics		-0.21
MLSE: Syntax		0.25
ACE-R: Visuospatial		0.15
**nfvPPA versus PSP and CBS**		
Model intercept	NA	-1.12
Stool		0.21
Looking		0.61
Sink		-0.33
Doing		0.54
Has/have		0.34
MLSE: Syntax		0.34
	**2. MLSE 'beach scene'**	
**Controls versus patients**		
Model intercept	-5.28	-9.04
Sandcastle	0.37	
Sun	1.88	1.88
Dig	1.12	
Book	0.37	
Sand	0.15	
Sea	0.59	

Wearing	2.30	1.34
Bone	1.26	0.49
Towel	0.32	0.56
Beach	0.75	0.86
MLSE: Syntax		0.03
MLSE: Working memory		0.11
ACE-R: Fluency		0.50
**"Motor" (nfvPPA, PSP, CBS) versus "Lexico-semantic" (svPPA, lvPPA)**		
Model intercept	-1.31	2.50
Sandcastle	-0.16	
Seagull	-0.96	-0.30
Do	1.22	0.44
Book	-0.35	-0.34
Know	1.31	1.64
Beach	0.40	0.38
Got	1.55	2.17
MLSE: Motor speech		0.02
MLSE: Semantics		-0.26
ACE-R: Fluency		-0.10
ACE-R: Visuospatial		-0.04
**svPPA versus lvPPA**		
Model intercept	NA	-1.54
Towel		-0.20
MLSE: Semantics		-0.22
MLSE: Syntax		0.43
ACE-R: Visuospatial		0.08
**nfvPPA and PSP versus CBS**		
Model intercept	NA	-0.29
Sandcastle		0.05
Sand		0.47
Towel		0.36
MLSE: Syntax		0.31
ACE-R: Visuospatial		-0.05

BDAE, Boston Diagnostic Aphasia Examination; CBS, corticobasal syndrome; LASSO, Least Absolute Shrinkage and Selection Operation; lvPPA, logopenic variant primary progressive aphasia; MLSE, Mini Linguistic State Examination; nfvPPA, non-fluent variant primary progressive aphasia; PSP, progressive supranuclear palsy; svPPA, semantic variant primary progressive aphasia.

Supplementary Table 9 A representative example of an anonymised svPPA patient using the BDAE 'cookie theft' 15-word checklist score sheet

**Step 1**: Listen for these words in the participant's narrative	**Step 2**: Score - was each word produced by the participant at least once? ✓ - yes ✗ - no	**Step 3**: Cross-out the points if the word was not produced	**Step 4**: Cross-out the points if the word was not produced
Doing	✗	~~8~~	n/a
Drying	✗	~~19~~	
Over	✗	~~3~~	
Garden	✗	~~5~~	
Overflow	✗	~~29~~	~~-15~~
Stool	✗	~~15~~	~~-11~~
Open	✗	~~19~~	~~-5~~
Sink	✗	~~4~~	~~-4~~
Not	✓	3	19
Water	✓	1	7
Something	✓	-6	2
Has/have	✓	n/a	16
Little	✓		11
Lady	✓		14
Looking	✗		n/a
**Step 5**: Sum the positive and negative "points" *only* for the words produced:		**Total score = __-2__**	**Total score = __69__**
**Interpretation**		~~Control vs~~ Patient	~~Motor vs~~ Lexico-Semantic Patient
		"Control" if the total score greater than 60 and "patient" if it is less than 60	"Lexico-semantic" if the total score is greater than 21 and "motor" if it is less than 21

BDAE, Boston Diagnostic Aphasia Examination; svPPA, semantic variant primary progressive aphasia.

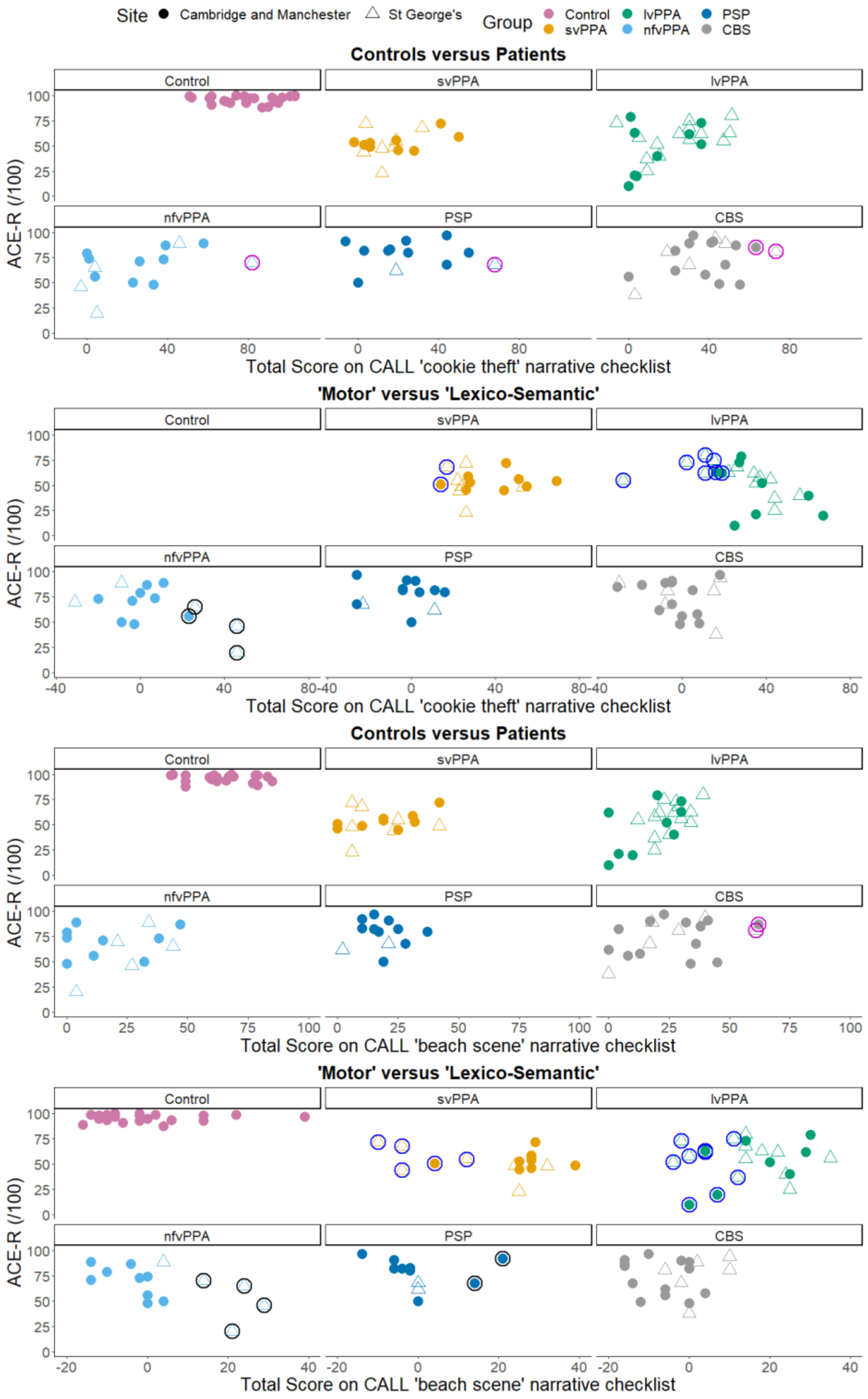

Supplementary Figure 1 Scatterplots showing total scores on the CALL 15-word checklists and ACE-R with the following color representations: magenta circles for people misclassified as controls, blue circles for those misclassified as belonging to the "motor" group, and black circles for those misclassified as belonging to the "lexico-semantic" group. ACE-R, Addenbrooke's Cognitive Examination – Revised; CALL, Cambridge Language List.

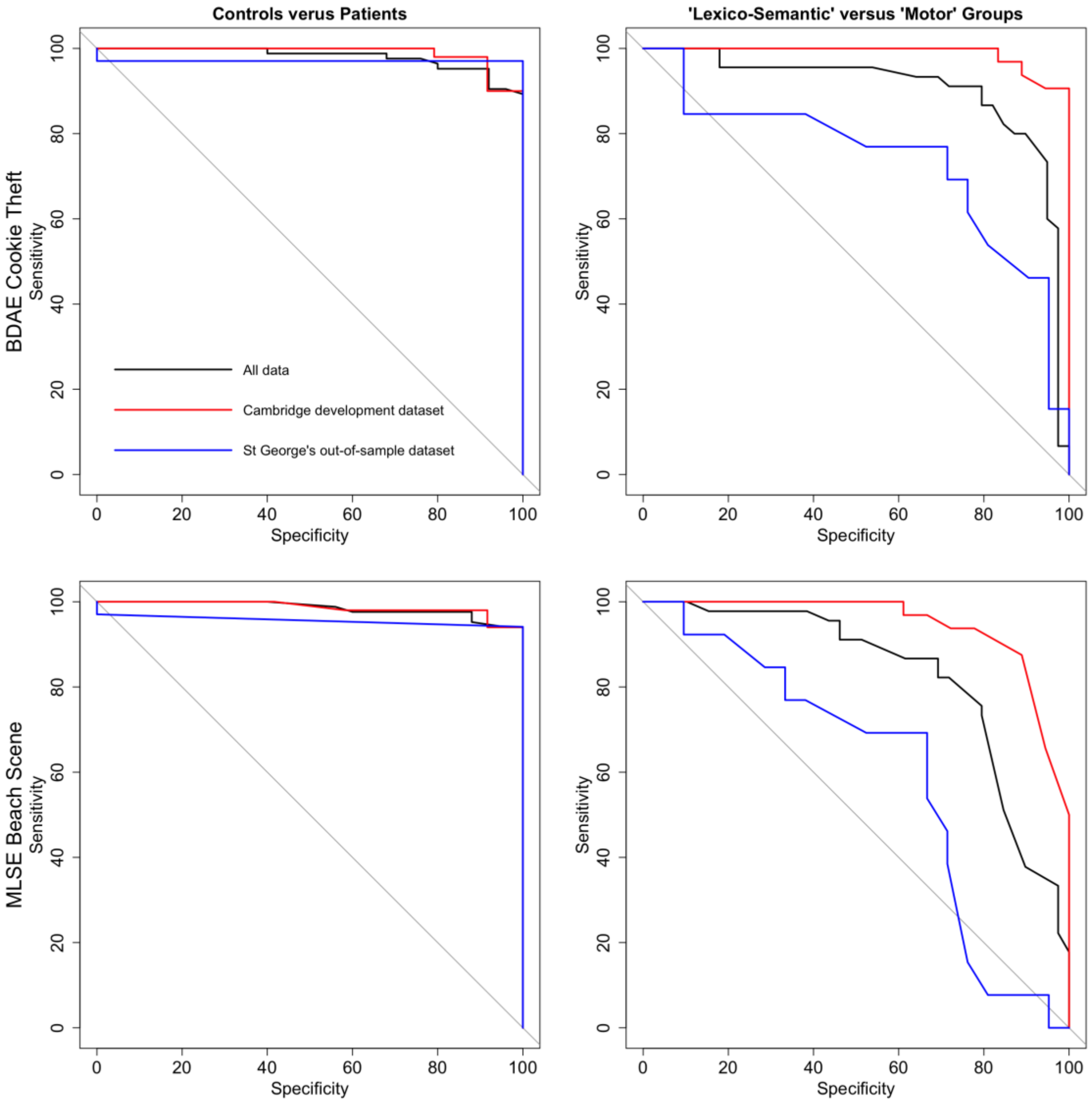

Supplementary Figure 2 Receiver operating characteristic (ROC) curves showing how well the CALL checklists distinguish between controls and patients (left), and between “lexico-semantic” and “motor” groups (right). All data are shown in black, the Cambridge validation dataset is shown in red, and St George’s out-of-sample dataset is shown in blue. When comparing controls relative to all patients using the BDAE ‘cookie theft’ checklist, the area under the curve (AUC) was largest for the Cambridge development dataset (98.92%), followed by all data (98%) and the St George’s out-of-sample dataset (97.06%). Using the MLSE ‘beach scene’ checklist, the AUC was largest for the Cambridge development dataset (98.67%), followed by all data (98.5%) and the St George’s out-of-sample dataset (95.59%). When comparing “lexico-semantic” versus “motor” groups using the BDAE ‘cookie theft’ checklist, the AUC was largest for the Cambridge development dataset (98.87%), followed by all data

(90.23%) and the St George's out-of-sample dataset (73.63%). Using the MLSE 'beach scene' checklist, the AUC was largest for the Cambridge development dataset (94.53%), followed by all data (82.65%) and the St George's out-of-sample dataset (60.07%). BDAE, Boston Diagnostic Aphasia Examination; CALL, Cambridge Language List; MLSE, Mini Linguistic State Examination.
